# Efficacy and safety of Chinese herbal medicine for pneumonia convalescence in children: A systematic review and meta-analysis

**DOI:** 10.3389/fphar.2022.956736

**Published:** 2022-09-02

**Authors:** Jian-Ning Guo, Xue Bai, Hong-Xian Zhang, Ning Zhang, Jun-Ming Liang, Zi-Yi Guo, Xia Cui

**Affiliations:** ^1^ Beijing University of Chinese Medicine Third Affiliated Hospital, Beijing, China; ^2^ School of Graduates, Beijing University of Chinese Medicine, Beijing, China

**Keywords:** Chinese herbal medicine, pneumonia convalescence in children, meta-analysis, systematic review, randomized controlled trials

## Abstract

**Background:** Chinese herbal medicine (CHM) has advantages in treating sequela symptoms of pediatric pneumonia convalescence. Hence, this study aims to evaluate the efficacy and safety of CHM using a meta-analysis approach.

**Methods:** The randomized controlled trials (RCTs) that met the search strategy were selected from seven databases from the inception date to December 17, 2021. Based on the Cochrane handbook, the quality of the selected studies was assessed using the risk of bias. Data were expressed as relative risk (RR) or mean difference (MD) and with 95% confidence interval (CI). Subgroup analyses and sensitivity analyses were performed. The Grading Recommendation Assessment, Development, and Evaluation (GRADE) method was used to assess the evidence certainty.

**Result:** Twenty RCTs with 2,241 participants were identified using the search criteria. CHMs included Danshen injection, Liujunzi decoction, Qingfei Tongluo decoction, Yiqi Huoxue decoction, Yupingfeng granule, XiaoErFeiKe granule, Sha-Sheng-Mai-Dong decoction, and so on. Results indicated that CHM combined with Western medicine (WM) or CHM alone improved the total clinical effective rate (RR = 1.22; 95% CI: 1.15–1.29), reduced cough relief time (MD = −2.16; 95% CI: −2.46 to −1.85), lung rales disappearance time (MD = −1.82; 95% CI: −2.17 to −1.47), and length of hospital stay (MD = −2.01, 95% CI: −3.81 to −0.22) in the treatment of pneumonia convalescence in children. However, there was no significant statistical difference regarding the incidence of adverse reactions (RR = 0.57; 95% CI: 0.23–1.43).

**Systematic Review Registration:** https://www.crd.york.ac.uk/PROSPERO/; Identifier CRD42022298936

## Graphical Abstract

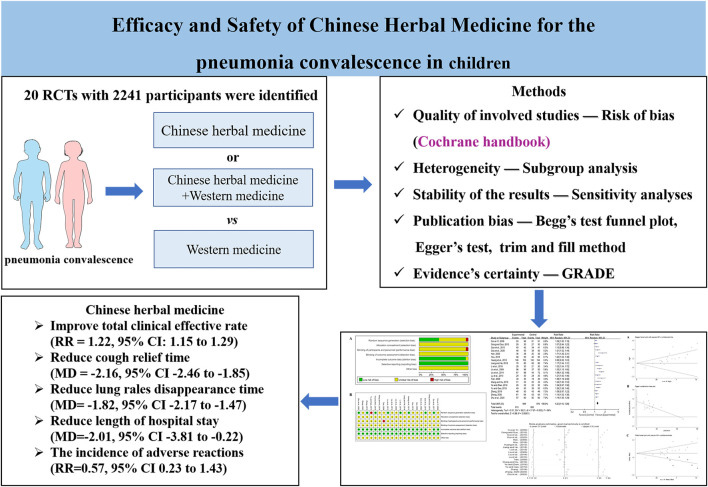

## 1 Introduction

Pneumonia is an inflammation of the lungs caused by different pathogens or other factors. In 2015, pneumonia caused the death of 920,000 children under the age of 5. Between 2000 and 2015, the number of hospitalizations caused by pneumonia in children increased by 187% and has posed a significant threat to society (Diseases and Injuries, 2020). Children are susceptible to various pathogenic microorganisms due to undeveloped airway cilia and immune systems (Quinton et al., 2018). Even after standard treatment, some children with pneumonia are still left with persistent cough, expectoration, wheezing, and other symptoms. Acute inflammation may gradually lead to chronic lung injury, which affects the normal development of lung tissue and lung function. This increases the risk of recurrent lower respiratory tract infections, asthma, bronchiolitis obliterans, and other diseases (Lee and Young Lee, 2020; Liu et al., 2021; Raita et al., 2021). Poor lung function affects the quality of life (Castro-Rodriguez et al., 1999; Chan et al., 2015; Perret et al., 2020). Frequent medical treatment and reduced quality of life cause a huge economic and social burden (Lewis et al., 2022). Hence, convalescent treatment of pneumonia is essential.

Antibiotics and hormone therapy have been used as the primary treatment strategies for pneumonia. However, the incidence of drug resistance has been increasing every year, resulting in reduced clinical efficacy. For viral pneumonia, immune inflammation damage, and persistent symptoms after pneumonia, related specific drugs for children are less. Clinical prevention and treatment are challenging (Gupta et al., 2018; Katz and Williams, 2018; Lee et al., 2018; Esposito et al., 2022). Hence, it is essential to identify new therapies for the treatment of pneumonia. Traditional Chinese medicine (TCM) has been shown to be beneficial for improving symptoms and eliminating viral infections, thus reducing hospitalizations during the pneumonia recovery period (Luo et al., 2020; Zhao et al., 2020; Chen et al., 2022). The characteristics of Chinese herbal medicine (CHM) have been shown to be a multi-component–multi-target–multi-pathway and have shown promise for the treatment of pneumonia (Lyu et al., 2021). *Huangdi Neijing*, an ancient and classic internal medicine book of TCM, described the lungs as a fragile organ that is easy to injure but difficult to heal (Lu, 1985). CHMs have been widely used for treatment of pediatric pneumonia, with promising outcomes. Pediatric pneumonia prevention and treatment studies have mainly focused on the acute or severe phase of pneumonia. Clinical studies have demonstrated the clinical efficacy of CHM in the treatment of acute or severe pneumonia (Ling et al., 2021; Peng et al., 2022). Nonetheless, more attention has been paid to the therapeutic effect in the recovery stage. CHMs that were reported with promising efficacy included Danshen injection, Liujunzi decoction, Yupingfeng granule, XiaoErFeiKe granule, and Sha-Sheng-Mai-Dong decoction. Additionally, there is a lack of evidence-based systematic reviews that have evaluated the safety and efficacy of CHM on pediatric pneumonia in convalescence. Hence, this study relied on the Cochrane systematic review method to compare the treatment of CHM combined with Western medicine (WM) or CHM alone with WM and evaluated the clinical efficacy and safety of CHM in convalescence children with pneumonia.

## 2 Methods

This systematic review followed the Preferred Reporting Items for Systematic reviews and Meta-Analyses (PRISMA) guidelines (Page et al., 2021). The protocol has been registered in PROSPERO (CRD42022298936).

### 2.1 Information sources and search strategy

The following databases were searched: China National Knowledge Infrastructure (CNKI), China Science and Technology Journal Database (VIP), Wanfang Data, Chinese Biomedical (CBM), PubMed, Embase, and Cochrane Library. The language of the publications was limited to Chinese and English. The publication date of retrieval was from the start of the corresponding database to December 11, 2021.

The search terms were “pneumonia convalescence,” “TCM or Chinese Traditional or Traditional Chinese Medicine or integrated Chinese and Western medicine,” “herbal medicine or injection or decoction or Chinese patent medicine,” “Random,” “randomized controlled trial,” and so on. Detailed search strategy and search results from the different databases are provided in Supplementary File S1.

### 2.2 Inclusion and exclusion criteria

Study type: The study design was a prospective, randomized controlled trial (RCT).

Participants: Patients below 18 years of age and who met the clinical diagnostic criteria for pneumonia convalescence.

Interventions: The intervention group: CHM or CHM combined with basic treatment of WM (unlimited dose, formula, and dosage form).

Comparators: The control group: WM, including anti-infection and relieving tracheal spasms, asthma, and cough.

Main outcomes: 1) Total clinical effective rate; 2) cough relief time; 3) lung rales disappearance time; 4) incidence of adverse reactions; and 5) length of hospital stay.

### 2.3 Exclusion criteria

The studies were not included if 1) duplicate conference publications were identified or were duplicated in Chinese or English (only the higher-quality publication was selected); 2) the primary outcome indicators were not included, or the data were insufficient to obtain; 3) the article types were case reports, case–control or cohort trials, meta-analyses or reviews, cell or animal experiments, and conference abstracts; and 4) the therapies included infantile massage, cupping, moxibustion, acupuncture, acupoint application, and other joint interventions.

### 2.4 Data extraction

Publications selected from the online databases were imported into document management software NoteExpress and Endnote X9. Publications were selected based on the inclusion and exclusion criteria by Jianning Guo and Xue Bai. Duplicate or unrelated publications were excluded based on titles and abstracts. After the initial screening, the full text was evaluated. Publications were then eliminated based on the exclusion criteria determined previously. Finally, the selected publications were used to extract the relevant information: 1) research characteristics, including publication year, name of the author, region, and funding sources; 2) the information of participants, such as cohort size, age, gender, course of the disease, and diagnostic criteria; 3) details of treatment, including different therapies, dosage, and duration; and 4) adverse events and outcome indicators.

### 2.5 Risk of bias assessment

The Cochrane bias risk rating scale was used to evaluate the methodological quality of the selected studies (Babic et al., 2019) and evaluated the following: correctness of the randomization method, covert grouping, the blind method, incomplete data, selective reporting, other deviations, etc. The risk assessment of bias was performed independently by two evaluators. Disagreements between the two evaluators were discussed with a third evaluator until a consensus was reached.

### 2.6 Strategy for data synthesis

RevMan 5.4 and Stata 15.0 software were used to analyze the data. Risk ratio (RR) with 95% confidence intervals (CIs) was used as an evaluation index for dichotomous data. Mean difference (MD) or standardized mean difference (SMD) with 95% CI was used as an evaluation index for continuous data. Heterogeneity was analyzed using the χ^2^ and *I* ^2^ tests. If there was no heterogeneity (*p* > 0.05, *I* ^2^ ≤ 50%), the fixed-effect model was selected; if there was heterogeneity (*p* ≤ 0.05, *I* ^2^ > 50%), the random-effect model was used. Potential sources of heterogeneity were further analyzed from the clinical, methodological, and statistical heterogeneity. Subgroup analysis was performed on the clinical characteristics to investigate the causes of clinical heterogeneity. By excluding one study at a time, sensitivity analysis was used to examine whether low-quality studies affected the stability of the overall meta-analysis (Wang et al., 2022). Only descriptive analysis was performed if heterogeneity could not be resolved.

### 2.7 Publication bias assessment

Begg’s test funnel plot and Egger’s test were used to evaluate publication bias. The impact of publication bias on the results was evaluated using the trim and filling method.

### 2.8 Evidence assessment

The Grading Recommendation Assessment, Development, and Evaluation (GRADE) system approach was used to evaluate the evidence level of each outcome index (Balshem et al., 2011). The risk of bias, result consistency, indirectness, accuracy, and publication bias of each outcome index were evaluated separately in GRADEpro (https://www.gradepro.org/). The quality of evidence was divided into four grades: high, medium, low, and very low.

## 3 Results

### 3.1 Study selection

**FIGURE 1:**
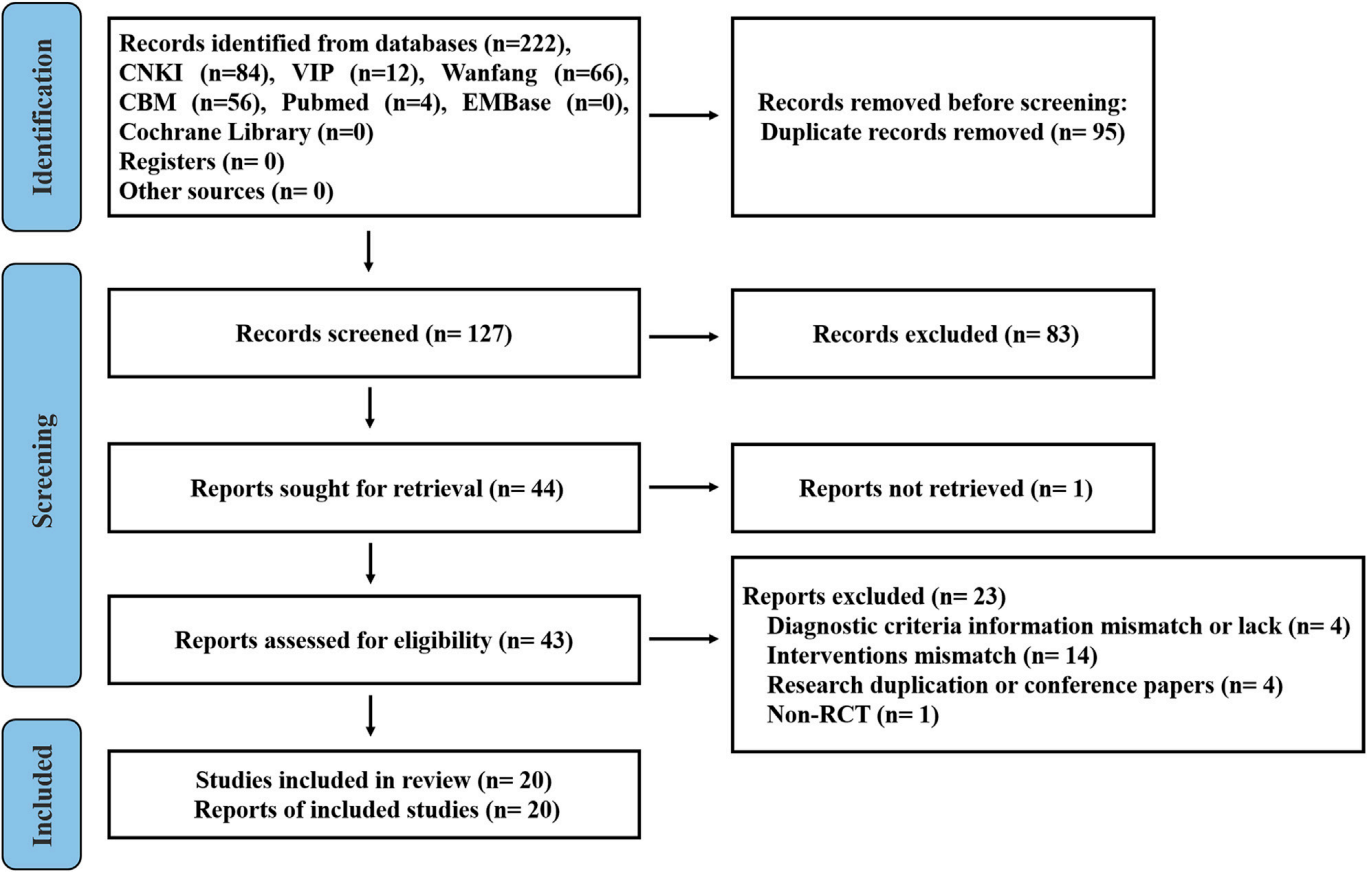
Flow diagram depicting the study selection process.

The study search strategy is shown in Figure 1. A total of 222 publications from the seven databases were retrieved. After eliminating 95 repeat publications, 127 publications were selected. Eighty-three publications were excluded after reviewing the titles and abstracts of the selected publications. One publication was not retrieved. After secondary assessment (reading the full text), 23 publications were excluded. Finally, 20 studies were selected that met the inclusion criteria.

### 3.2 Study characteristics

Table 1 shows the baseline information of all the selected studies. These studies were of Chinese patients and published in Chinese language journals from 2009 to 2020. A total of 2,241 children were enrolled in these 20 studies (Cui and Yin, 2009; Han, 2009; Liu, 2009; Tian, 2009; Gong and Guo, 2010; Yu and Gao, 2010; Huang et al., 2013; Lu et al., 2013; Liu et al., 2014; Guo et al., 2015; Hou, 2016; Ye and Zeng, 2016; Du et al., 2017; Liang and He, 2018; Zhang, 2018; Li and Cai, 2019; Wang and Hu, 2019; Guo et al., 2020; Zhang, 2020; Zhu et al., 2020), which included 1,161 patients in the intervention groups and 1,080 in the control groups. Diagnostic criteria included *Zhufutang Practical Pediatrics*, *Internal medicine*, and self-prepared. The treatment period was 3–14 days.

**TABLE 1:** Baseline characteristics of the selected studies for meta-analysis.

Study ID	Region	Funding source	Sample size (T/C)	Age (Y) (T/C)	Gender (M/F)	Diagnosis standard	Intervention		Duration (days)	Adverse effect		Management for patients with AEs	Outcome
							T	C (ST)		T	C		
Cui and Yi, (2009)	China	NR	187 (96/91)	3m–3y	109/78	*Zhufutang Practical Pediatrics*	Liujunzi decoction (infants, 50 ml; baby, 100 ml; young children, 150 ml; school-age children, 200 ml, drink daily at any time po) + ST	1 or 2 antibiotics (usually β-lactams or macrolides) for anti-infection, α-chymotrypsin, and dexamethasone for nebulized inhalation (NR)	3d	NR	NR	NR	1
Du et al. (2017)	China	Science and Technology Project of Hebei Province	120 (60/60)	(8.44 ± 1.34)/(7.35 ± 1.35)	65/55	*Zhufutang Practical Pediatrics*	Yupingfeng granule (≤3y, 2.5 g, tid; >3y, 5 g, tid po) + ST	Spleen aminopeptide lyophilized powder (2 mg/time, qd, po) and symptomatic treatment (NR)	14d	0	0	AEs were tolerable and did not affect treatment	2.3
Gong and Guo, (2010)	China	NR	60 (30/30)	(5.6)/(5.4)	33/27	*Zhufutang Practical Pediatrics*	Self-made formula (bid, po) + ST	Cefoperazone/sulbactam sodium (80 mg/kg, qd, ivgtt); ambroxol (15mg, qd, ivgtt); ambroxol hydrochloride and clenbuterol hydrochloride oral solution (NR)	7d	NR	NR	NR	1
Guo et al. (2015)	China	NR	83 (42/41)	(1.5 ± 2.5)/(1.3 ± 2.5)	44/39	*Zhufutang Practical Pediatrics*	Self-made formula (bid, po) + ST	Latamoxef sodium injection (60 mg/kg, bid, ivgtt), ambroxol injection (15 mg/kg, qd, ivgtt), and ambroxol (15 mg/kg, bid, nebulized inhalation)	3–5d	NR	NR	NR	1
Guo et al. (2020)	China	Project of Traditional Chinese Medicine Bureau of Guangdong Province	100 (50/50)	(5.59 ± 2.23)/(5.62 ± 2.14)	61/39	*Internal medicine*	Qingfei Tongluo decoction (NR) + ST	Azithromycin (10 mg/kg, tid, po); sulbactam sodium injection (50 mg/kg, qd, ivgtt); ambroxol hydrochloride injection (7.5 mg, tid, ivgtt)	NR	NR	NR	NR	1
Han, (2009)	China	NR	74 (38/36)	9m–14y/8m–14y	39/35	NR	Sha-Sheng-Mai-Dong decoction (tid, po)	MA (no details)	7–14d	NR	NR	NR	1
Hou, (2016)	China	NR	100 (50/50)	(1.12 ± 0.23)/(1.32 ± 0.19)	54/46	NR	Liujunzi decoction (NR)	Ampicillin, penicillin, cephalosporins (po or ivgtt, NR), dexamethasone (inh, NR)	3d	NR	NR	NR	1
Huang et al. (2013)	China	Guangxi Scientific Research and Technology Development Program	296 (152/144)	1–6y	155/141	*Convalescent period of pneumonia in children (self-prepared)*	Self-made formula (tid, po) + ST	Cefoperazone sodium and sulbactam sodium (100 mg/kg, bid, ivgtt) and symptomatic treatment (NR)	7d	NR	NR	NR	1.5
Liang and He, (2018)	China	NR	160 (80/80)	(6.0 ± 1.2)/(5.8 ± 2.0)	86/74	*Zhufutang Practical Pediatrics*	Huoxuehuayu decoction (bid, po) + ST	Azithromycin (10 mg/kg, qd, ivgtt) and atomization or symptomatic treatment (NR)	10d	0	0	AEs were tolerable and did not affect treatment	1
Li and Cai, (2019)	China	NR	60 (30/30)	(4.64 ± 1.23)/(4.42 ± 1.13)	31/29	*Zhufutang Practical Pediatrics*	XiaoErFeiKe granule (<1 y, 2 g; 1–4 y, 3g; 5–8y, 6 g, tid, po) + ST	Ceftazidime sodium injection (30–100 mg/kg, bid-tid, ivgtt)	7d	Two cases of diarrhea	One case of diarrhea and one case of nausea	AEs were tolerable and did not affect treatment	1,2,3,4
Liu., 2009	China	NR	146 (96/50)	(3.1 ± 2.6)/(3.2 ± 2.8)	76/70	*Zhufutang Practical Pediatrics*	Addition of six Junzi decoction (tid, po)	ST (NR)	10d	NR	NR	NR	1.5
Liu et al. (2014)	China	NR	160 (88/72)	3m–12y	NR	*Zhufutang Practical Pediatrics*	Yiqi Jianpi Huoxue decoction (NR) + ST	ST (NR)	7d	NR	NR	NR	1
Lu et al. (2013)	China	NR	60 (30/30)	(3.3 ± 1.84)/(3.8 ± 2.24)	34/26	*Zhufutang Practical Pediatrics*	Addition of Yupingfeng oral liquid and Xingbei expectorant cough oral liquid (<1 y, 5 ml/time; 1–6y, 10 ml/time; 6–10y, 15ml/time; ≥10 y, 20 ml/time, tid)	Pidotimod granules (0.4 g, bid, po), ambroxol hydrochloride and clenbuterol hydrochloride oral solution (NR)	7d	NR	NR	NR	1
Tian, (2009)	China	NR	60 (30/30)	(3.29 ± 1.83)/(2.67 ± 2.34)	34/26	*Zhufutang Practical Pediatrics*	Yiqi Huoxue decoction (bid, po) + ST	Anti-infection and phlegm treatment (NR)	3d	0	0	AEs were tolerable and did not affect treatment	1.5
Wang and Hu, (2019)	China	NR	66 (33/33)	(4.56 ± 1.98)/(5.89 ± 2.18)	32/34	NR	XiaoErFeiKe granule (3–8g, tid, po) + ST	Ambroxol hydrochloride injection (30 mg/time, qd, atomized inhalation) and cefamandole nafate for iInjection (50–100 mg/kg, qd, ivgtt)	14d	One case of diarrhea	one case of diarrhea and one case of nausea	AEs were tolerable and did not affect treatment	1,2,3,4
Ye and Zhen, (2016)	China	NR	62 (32/30)	(4.6 ± 1.4)/(4.5 ± 1.2)	33/29	*Zhufutang Practical Pediatrics*	XiaoErFeiKe granule (<1 y, 2 g; 1–4 y, 3 g; 5–8y, 6 g, tid, po) + ST	Cefamandole nafate for injection (50–100 mg/kg, bid, ivgtt) and ambroxol injection (30 mg nebulized inhalation, qd)	14d	One case of diarrhea and one case of nausea	Two cases of diarrhea	AEs were tolerable and did not affect treatment	1,2,3,4
Yu and Gao, (2010)	China	NR	200 (100/100)	2m–3y	110/90	*Zhufutang Practical Pediatrics*	Danshen injection (4–8 ml, bid, ivgtt) + ST	Erythromycin (100 mg/kg, bid, ivgtt); cefuroxime sodium (50 mg/kg, bid, ivgtt); Mucosolvan pump inhalation (7.5 mg, qd, inh)	7d	NR	NR	NR	1,2,3
Zhang, (2018)	China	NR	99 (50/49)	(3.71 ± 1.64)/(4.01 ± 1.93)	48/51	*Zhufutang Practical Pediatrics*	Lin gui zhi ke combination (1–3y, 15 m1/time; 3–7y, 30 m1/time; 7–12y, 50m1/time, tid, po)	Sequential therapy: 1. Penicillin + amoxicillin and clavulanate potassium granules (1–7y, 0.15625g/time, tid; 7–12y, 0.15625g*1.5/time, tid, po) + ambroxol hydrochloride syrup (1–2y, 2.5m1/time, bid; 2–6y, 2.5m1/time, tid; 6–12y, 5ml/time, bid or tid, po); 2. cephalosporins + cefaclor for suspension (20–40 mg/kg, tid, po) + ambroxol hydrochloride syrup	7d	NR	NR	Two cases of mild diarrhea and associated with drinking cold. AEs were tolerable and did not affect treatment	1.5
Zhang, (2020)	China	NR	128 (64/64)	(5.2 ± 2.3)/(5.3 ± 2.7)	74/54	NR	Erchen decoction or Xiaoqinglong decoction (<1y, 5ml; >1y, 10ml, tid, po) + ST	Anti-infection (NR), ambroxol and budesonide (NR)	5d	NR	NR	NR	1
Zhu et al. (2020)	China	NR	120 (60/60)	(8.12 ± 2.07)/(8.08 ± 1.98)	56/64	*Zhufutang Practical Pediatrics*	Ren Shen Wu Wei Zi decoction or Sha-Sheng-Mai-Dong decoction (1–3y, 5 ml/time; 4–7y, 10 ml/time; 7–12y, 12 ml/time, tid, po)	Ambroxol hydrochloride oral solution (1–2y, 2.5 ml/time, bid; 2–5y, 2.5 ml/time, tid; 6–12 y, 5ml/time, bid)	10d	One case of diarrhea	One case of emesis, two cases of diarrhea, three cases of indigestion, one case of anaphylaxis	NR	1,2,3,4,5

Note: AE, adverse effects; bid, twice a day; C, control group; d, day; F, female; inh, inhalation; ivgtt, intravenous drip; M, male; MA, macrolide antibiotics; m, month; NR, not reported; po, per os; qd, quaque die (once a day); ST, symptomatic treatment; T, treatment group; tid, three times a day; y, year; 1, total clinical effective rate; 2, cough relief time; 3, lung rales disappearance time; 4, the incidence of adverse reactions; 5, length of hospital stay.

Fifteen studies compared CHM plus WM with WM, and five studies compared CHM alone with WM. CHMs that were used included Danshen injection, Liujunzi decoction, Qingfei Tongluo decoction, Yiqi Huoxue decoction, Yupingfeng granule, XiaoErFeiKe granule, Sha-Sheng-Mai-Dong decoction, etc. The detailed composition of the CHMs used is provided in Supplementary File S2. Conventional symptomatic treatment drugs included azithromycin, erythromycin, cefuroxime sodium, cefoperazone/sulbactam sodium, ambroxol, etc.

### 3.3 Risk of bias for inclusion in the trial

**FIGURE 2:**
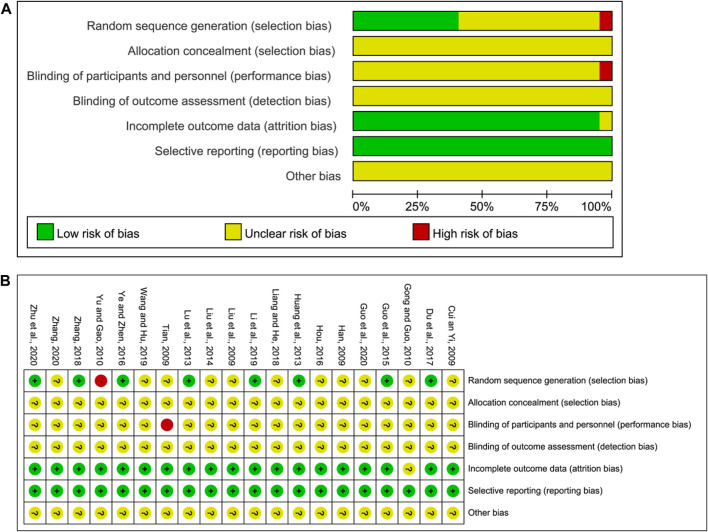
**(A)** Risk of bias graph and **(B)** risk of bias summary.

Two of the authors in this study independently assessed the risk of bias. The assessment results are shown in Figure 2. All studies described the randomized allocation, while eight studies reported on the generation of random sequences by the random number table, which was considered low risk. Yu and Gao (Yu and Gao, 2010) reported the randomization by order of admission, and this was considered high risk. The remaining studies did not elaborate on the random sequence information. As for selection bias, no study mentioned hidden allocation information and hence was considered unclear. As for the risk of performance and detection bias, Tian (Tian, 2009) divided the study groups based on the hospital admission order and hence were considered high risk. The remaining studies did not report the blinding method, and hence the risk of performance and detection bias was unclear. Gong and Guo (Gong and Guo,2010) did not report sufficient outcome data and hence were rated as unclear. The remaining studies published all dates, and hence the risk of reporting bias was rated as low. The risk of other biases was unclear and hence insufficient to make a judgment.

### 3.4 Meta-analysis outcomes

#### 3.4.1 Total clinical effective rate

**FIGURE 3:**
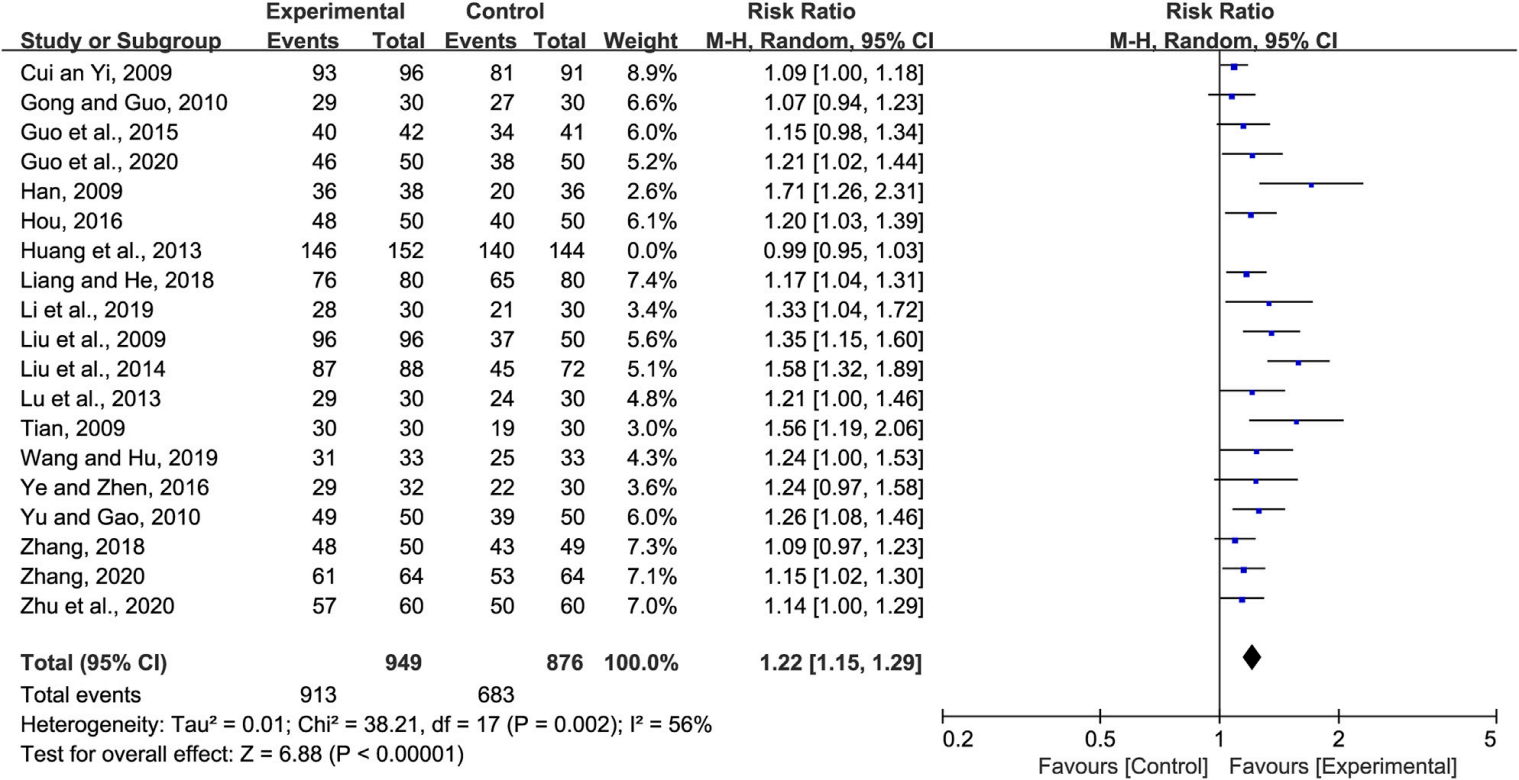
Forest plot for the total clinical effective rate.

Nineteen studies reported the total clinical effective rate of CHM for the treatment of pneumonia convalescence in children; however, heterogeneity was observed (*p <* 0.0001 and *I* ^*2*^ = 83%). Hence, sensitivity analyses were performed by excluding studies, one at a time (Wang et al., 2022). Heterogeneity was significantly reduced after eliminating the study by Huang et al. (Huang et al., 2013), (*p* = 0.002 and *I* ^*2*^ = 56%). The self-made diagnostic criteria by the authors were the main reason for its clinical heterogeneity. As a result, the random-effects model was selected to perform the meta-analysis. Our analysis indicated that the total clinical effective rate of the experimental group (CHM + WM and CHM alone) was significantly better than that of the control group (WM) (RR = 1.22, 95% CI 1.15 to 1.29, *p <* 0.00001, Figure 3). Subgroup analyses were performed based on the therapy method, treatment course, efficacy judgment standards, and diagnostic criteria (Supplementary File S3). We found that the 14-day course was more effective (RR = 1.35 95% CI 1.11 to 1.64, *p =* 0.003). These factors did not influence the results, and sensitivity analysis indicated the results to be stable (Supplementary File S4).

#### 3.4.2 Cough relief time

**FIGURE 4:**
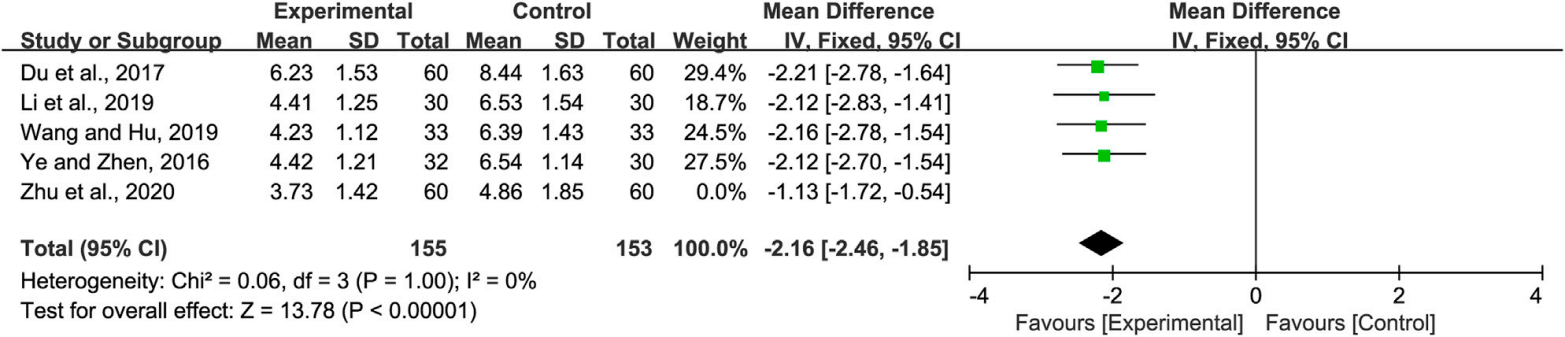
Forest plot for cough relief time.

Five studies reported on the cough relief time. We found significant heterogeneity (*p =* 0.06 and *I* ^*2*^ = 57%). Sensitivity analyses were performed, and after excluding the study by Zhu et al. (Zhu et al., 2020), heterogeneity between the studies was significantly reduced (*p* = 1 and *I* ^2^ = 0%). Hence, the fixed-effects model was selected. We found that cough relief time in the experimental group was shorter than that of the control group (MD = −2.16, 95% CI −2.46 to −1.85, *p <* 0.00001, Figure 4). Subgroup analysis was not performed due to the limited number of studies available.

#### 3.4.3 Lung rales disappearance time

**FIGURE 5:**
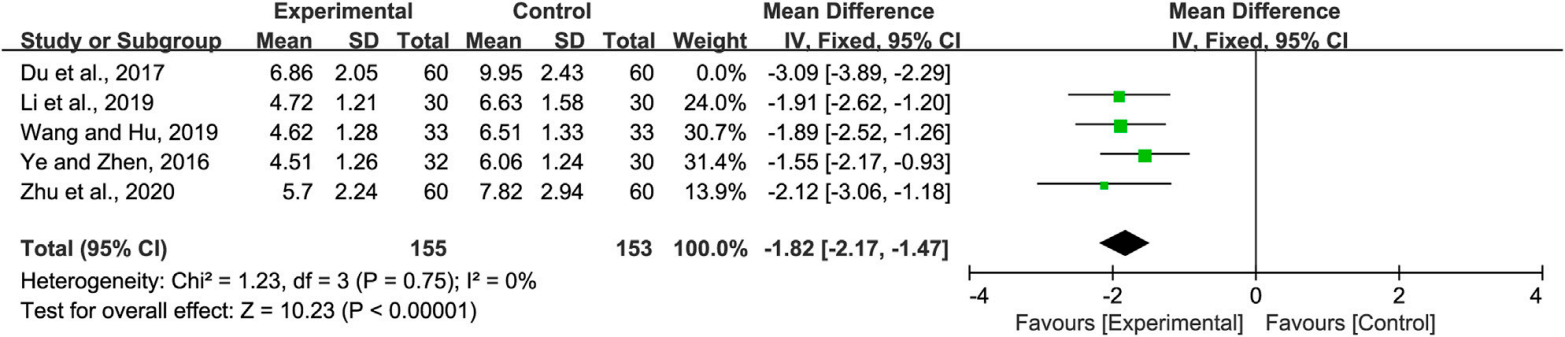
Forest plot for lung rales disappearance time.

Five studies included information regarding lung rales disappearance time. Significant heterogeneity was observed (*p =* 0.05 and *I* ^*2*^ = 57%). Sensitivity analyses were performed, and after excluding the study by Du et al. (Du et al., 2017), the heterogeneity between the studies was significantly reduced (*p =* 0.56 and *I* ^*2*^ = 0%). Hence, the fixed-effects model was selected. Our analysis showed that the lung rales disappearance time in the experimental group was shorter than that of the control group (MD = −1.82, 95% CI −2.17 to −1.47, *p <* 0.00001, Figure 5). Subgroup analysis was not performed due to the limited number of studies available.

#### 3.4.4 Incidence of adverse reactions

**FIGURE 6:**
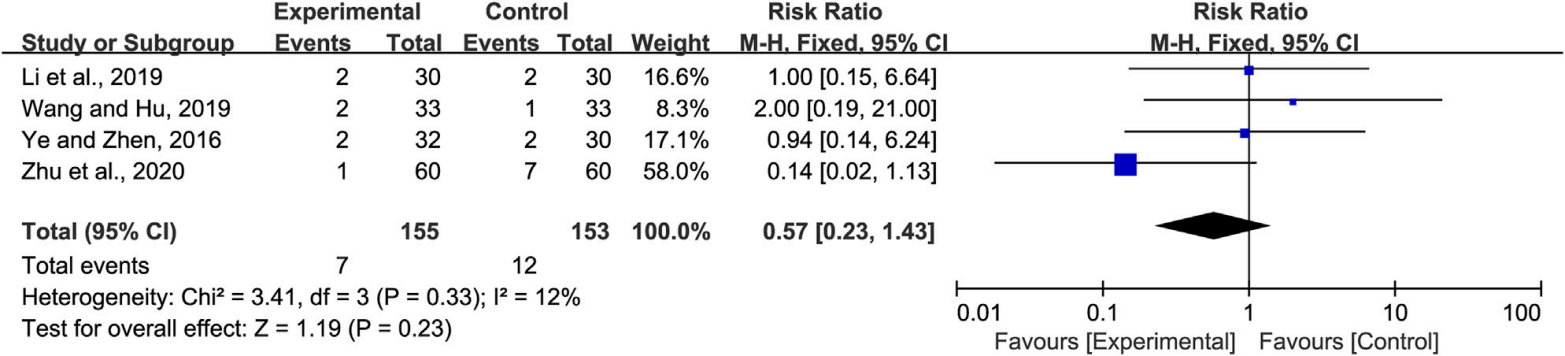
Forest plot for adverse reactions incidence.

Four studies included the incidence of adverse reactions. Li and Cai (Li and Cai, 2019) reported two cases of diarrhea in the experimental group, while one case of diarrhea and one case of nausea were observed in the control group. Wang and Hu (Wang and Hu, 2019) reported one case of diarrhea in the experimental group, while there was one case of diarrhea and one case of nausea in the control group. Ye and Zeng (Ye and Zeng, 2016) reported one case of diarrhea and one case of nausea in the experimental group, while there were two cases of diarrhea in the control group. Zhu et al. (Zhu et al., 2020) reported one case of diarrhea in the experimental group, while there was one case of emesis, two cases of diarrhea, three cases of indigestion, and one case of anaphylaxis in the control group. All adverse effects (AEs) were tolerable and did not affect treatment. Meta-analysis indicated little heterogeneity (*p =* 0.33 and *I* ^*2*^ = 12%). Hence, the fixed-effects model was selected for meta-analysis. The analysis of all the studies indicated no obvious significance in the incidence of adverse reactions between the experimental and the control group (RR = 0.57, 95% CI 0.23 to 1.43, *p =* 0.23, Figure 6).

#### 3.4.5 Length of hospital stay

**FIGURE 7:**
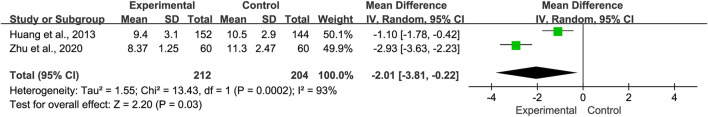
Forest plot for hospital stay length.

Two studies reported the length of hospital stays. Analysis indicated that there was significant heterogeneity (*p =* 0.0002 and *I* ^*2*^ = 93%). Hence, the random-effects model was selected. Our analysis showed that compared with the control group, the length of hospital stays for the experimental group was shorter (MD = −2.01, 95% CI −3.81 to −0.22, *p =* 0.03, Figure 7). Sensitivity analyses and subgroup analyses were not performed due to the limited number of studies available.

### 3.5 Publication bias

**FIGURE 8:**
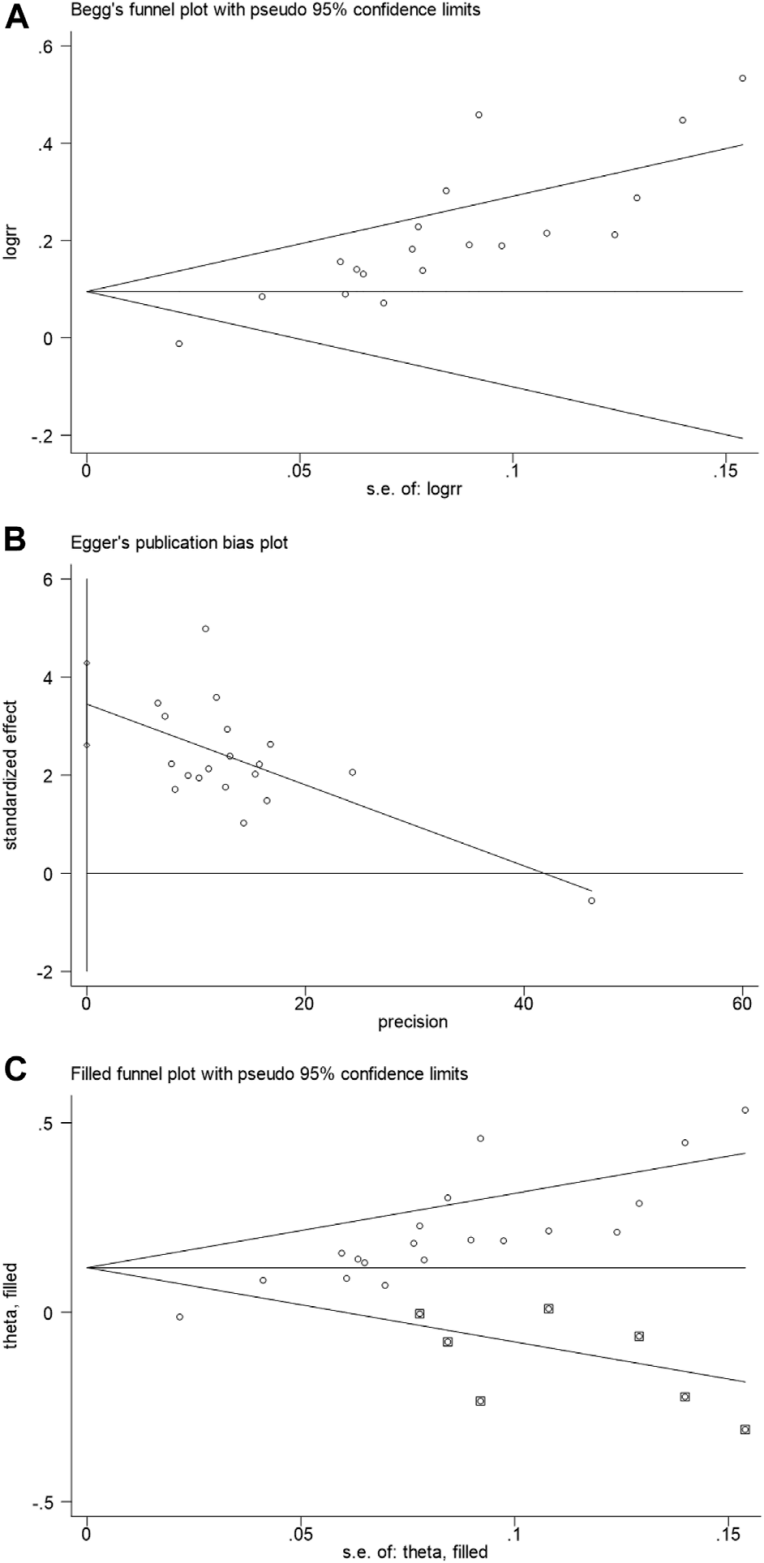
**(A)** Begg’s test funnel plot; **(B)** Egger’s test; and **(C)** filled funnel plot.

Begg’s test funnel plot and Egger’s test were used to evaluate the publication bias of the total clinical effective rate. A value of *p* = 0.002 for Begg’s test and *p* < 0.001 for Egger’s test suggested the presence of publication bias (Figures 8A,B). Subsequently, the trim and fill method was performed. We observed before fill (RR: 0.186; 95% CI: 0.112, 0.250; *p* < 0.001) and after fill for the seven studies (RR: 0.118; 95% CI: 0.058, 0.178; *p* < 0.001, Figure 8C). Hence, the meta-analysis results were considered robust.

### 3.6 Certainty assessment

After sensitivity analysis and excluding studies that affected heterogeneity, GRADE was performed to assess evidence certainty. All outcomes were evaluated as moderate certainty to very low certainty, as shown in Table 2. The reasons for downgrading were risk of bias, significant heterogeneity-induced inconsistency, publication bias, and small sample size, resulting in inaccuracy.

**TABLE 2:** Quality of evidence by GRADE.

Outcome	Anticipated absolute effects* (95% CI)		Relative effect (95% CI)	No. of participant (study)	Certainty of the evidence (GRADE)	Comment
	Risk with WM	Risk with CHM				
Total clinical effective rate	780 per 1,000	951 per 1,000	RR 1.22 (1.15–1.29)	1825 (18 RCTs)	⊕○○○ Very low^a^ ^,^ ^b^ ^,^ ^c^	Risk of bias (-1) Inconsistency (-1) Publication bias (-1)
Cough relief time	The mean cough relief time was 0	MD 2.16 lower (2.46 lower to 1.85 lower)	—	308 (4 RCTs)	⊕⊕⊕○ Moderate^a^	Risk of bias (-1)
Lung rales disappearance time	The mean lung rales disappearance time was 0	MD 1.82 lower (2.17 lower to 1.47 lower)	—	308 (4 RCTs)	⊕⊕⊕○ Moderate^a^	Risk of bias (-1)
Incidence of adverse reactions	78 per 1,000	45 per 1,000 (18–112)	RR 0.57 (0.23–1.43)	308 (4 RCTs)	⊕⊕○○ Low^a^ ^,^ ^d^	Risk of bias (-1) Imprecision (-1)
Length of hospital stay	The mean length of hospital stay was 0	MD 2.01 lower (3.81 lower to 0.22 lower)	—	416 (2 RCTs)	⊕○○○ Very low^a^ ^,^ ^b^ ^,^ ^d^	Risk of bias (-1) Inconsistency (-1) Imprecision (-1)

Notes: MD, mean difference; RCTs, randomized controlled trials; risk ratio (RR).

a Most indexes in included studies are at low or unclear risk of bias, while the individual study was at high risk in randomization process and blinding.

b There was heterogeneity and I^2^ ≥ 75%.

c Asymmetric funnel plot.

d Small sample size.

## 4 Discussion

### 4.1 Primary results

This study included 20 RCTs for meta-analysis to assess the efficacy and safety of CHM in combination with WM or CHM alone with that of WM for the treatment of pediatric pneumonia convalescence. Our analyses demonstrated that CHM had the advantage of enhancing therapeutic efficacy and reducing the number of cough days, lung rales disappearance time, and hospitalization time compared with patients who were in the WM treatment group. In subgroup analyses, CHM combined with WM and CHM alone could increase the total effective rate. The results were not influenced by the different treatment courses, diagnostic methods, and efficacy standards. Regarding the incidence of adverse events, no adverse events were observed in three studies, while four studies described adverse events; there was no significant difference between CHM combined with WM or CHM alone and WM. All adverse reactions, such as diarrhea, nausea, emesis, indigestion, and anaphylaxis, were mild for all groups and did not influence therapy.

Some children still have persistent cough, expectoration, wheezing, and other symptoms after pneumonia. More than one-third of children with pneumonia are susceptible to being hospitalized for disease relapse or deterioration within 1 week after discharge. The long and frequent hospitalizations cause a substantial economic and social burden (Neuman et al., 2014; Lewis et al., 2022). We found that CHM combined with WM or CHM alone could reduce the cough relief time (MD: −2.16 day, 95% CI: −2.46, −1.85) as well as lung rales disappearance time (MD: −1.82 day, 95% CI: −2.17, −1.47). In addition, CHM reduced the length of hospital stays (MD: −2.01 day, 95% CI: −3.81, −0.22) and was found to reduce symptoms during the convalescent period of pneumonia and hence reduce the financial burden and missed work and school time brought by hospitalization for both the child and the family.

Two publications performed follow-up studies for recurrence of infection in pneumonia convalescence children. Huang et al. (Huang et al., 2013) found that in 152 children in the CHM + WM group, 24 children had a recurrence, with 31 infections during the 1 month after discharge. In contrast, among the 144 children in the WM group, 45 children had a recurrence, with 68 infections during the 1 month after discharge. Han (Han, 2009) established the efficacy criteria for relapse time and found that, among the 38 children who received CHM alone, 13 cases had recurrence within 1–6 months after treatment, with a total effective rate of 65.8% (infection recurred within 1 year or 6 months after treatment); while, among the 36 children in the WM group, 24 cases had recurrence within 1–6 months, with the total effective rate of 33.3% after treatment. These results indicated that the CHM combined with WM or CHM alone could reduce the risk of recurrent respiratory infection and pneumonia.

Guo et al. (Guo et al., 2020) reported on the changes in lung function before and after treatment. Vital capacity (VC), forced expiratory volume in the first second (FEV1), and maximal voluntary ventilation (MVV) were lower than 60% before treatment but improved after treatment. CHM combined with WM was more effective in improving lung function than WM. Long-term clinical studies had shown that children who were diagnosed with pneumonia or lower respiratory tract infections before the age of 3 had an impact on their lung function in school-age adolescents, and even adults. Low levels of FEV 1 and forced expiratory flow (FEF) 25–75 and small airway obstructions were observed in these children. Follow-up studies found that the susceptibility to lower respiratory tract infections increased during childhood, and sequelae such as asthma and chronic obstructive pulmonary disease (COPD) risk increased in adulthood (Castro-Rodriguez et al., 1999; Chan et al., 2015; Perret et al., 2020). There are only a few studies that have been performed on the effectiveness of CHM in pulmonary function recovery and follow-up studies on pneumonia convalescence children. Additional clinical studies are required to address this limitation.

### 4.2 Potential molecular mechanism

CHMs are multiple component compounds that regulate multiple metabolic pathways. It has been shown to improve acute and chronic inflammatory injury induced by pneumonia. It has also been shown to regulate the immune system and promote lung tissue repair (Ding and Liu, 2019; Xi et al., 2020). Several studies performed on Chinese herbal compounds have been summarized in Table 1. Liujunzi decoction has been shown to block the phosphorylation of IκB-α and nuclear factor kappa-B (NF-κB), enhance the activity of antioxidant enzymes, and reduce lipid oxidation levels, while simultaneously inhibiting the secretion of inflammatory cytokines such as TNF-α, IL-1β, and IL-6 to protect against lung injury in cigarette smoke-induced COPD mouse models (Zhou et al., 2016). Naringenin is an important component of the Qingfei Tongluo formula. It has been shown to inhibit autophagy-mediated inflammatory cytokines and reduce *mycoplasma pneumoniae* in pneumonia-induced lung injury and pulmonary fibrosis mouse models (Lin et al., 2018). Sha-Shen-Mai-Dong decoction has been shown to increase IFN-γ levels and reduce IL-4 levels, regulate Th1/Th2 immune imbalance, and improve pathological lung injury in rat models (Yang and Zhou, 2019). Yupingfeng powder has been shown to have a regulatory effect on the immune system. It has been shown to alleviate asthmatic inflammatory cell infiltration and mucus secretion in mouse models by blocking NOD-like receptor thermal protein domain associated protein 3 (NLRP3) inflammasomes and alleviating pneumonia inflammation (Liu et al., 2017; Li et al., 2022). Furthermore, more studies have demonstrated the efficacy of CHM and deciphered the mechanism of action. In our meta-analysis, we found that the most frequently used CHMs were *Radix Glycyrrhizae*, *Codonopsis affinis Hook. f. and Thomson*, *Poria*, *Arum ternatum Thunb.*, *Hedysarum Multijugum Maxim.*, *Pericarpium Citri Reticulatae*, and *Radix Salviae liguliobae.* Understandably, the number of pharmacological studies of these CHMs is increasing due to their effectiveness in alleviating pneumonia convalescence in children.

### 4.3 Implications

For the clinical treatment of pneumonia convalescence in children, CHM combined with WM or CHM alone could improve the therapeutic effect, accelerate the recovery of cough and lung rales, and reduce the length of hospital stay, with a favorable safety profile. The mechanisms of CHMs in regulating immune function, downregulating inflammation, and promoting lung tissue repair have also been gradually clarified (Xi et al., 2020; Lyu et al., 2021). Hence, this meta-analysis provides a reference value for clinical treatment. Moreover, the diagnosis and treatment of pneumonia convalescence need to be standardized. Whether the combination of CHM could reduce the use of WM such as antibiotics is worth exploring.

At the same time, the limited evidence indicated that CHM could improve lung function and reduce the recurrence of respiratory infections. Critical long-term outcomes, follow-up, and lung function are rarely reported. Clinical investigators should register their protocol in advance and improve the quality of clinical research.

### 4.4 Strengths and limitations

This study has filled the gaps in evaluating the efficacy and safety of CHM in the treatment of pediatric pneumonia convalescence and provided reference evidence for clinical treatment guidelines. Two researchers with good evidence-based medicine training predefined and published systematic and comprehensive retrieval strategy and screening criteria. Moreover, the risk of bias, heterogeneity, publication bias, and evidence quality were evaluated by different methods.

As for limitations, heterogeneity is a problem for processing data in systematic reviews. We found that the current treatment strategies for pediatric pneumonia during the convalescent stage were mainly based on syndrome differentiation, which indicates that patients showing different syndromes were administered different drugs. This may lead to clinical heterogeneity. We tried to reduce clinical heterogeneity based on clinical characteristics (Higgins et al., 2003; Ioannidis et al., 2008). The severity of pneumonia in children, different therapies, treatment courses, evaluation criteria, and whether it was based on syndrome differentiation of TCM were the potential reasons for clinical heterogeneity (Melsen et al., 2014; Ling et al., 2021). Subgroup and sensitivity analyses revealed that different subgroup factors were not the cause of heterogeneity. Notably, the incidence of residual symptoms in pneumonia convalescence might impact heterogeneity. We did not evaluate it due to limited information. The heterogeneity of the incidence among different ages, pathogenic types of pneumonia, region, and ethnicity still needs to be considered. Sensitivity analyses were performed using Stata 15.0 and indicated that the results were reliable. In addition, most studies were small trials and failed to adequately provide information on allocation concealment and blind random allocation, resulting in risk of bias. This may have led to exaggerated treatment effects and methodological heterogeneity (Higgins et al., 2003; IntHout et al., 2015).

Regarding publication bias, Begg’s funnel plot of the total clinical effective rate is asymmetric. Most studies failed to mention clinical trial protocol registration information and study quality control. Our meta-analysis results were robust after the seven studies underwent the trim-and-fill method. The absence of gray and negative studies may have contributed to publication bias. Disclosure of negative results in clinical studies is important to demonstrate clinical relevance. Moreover, the majority of the included studies were small-sample trials and could have been vulnerable to selection bias, while large-sample trials were relatively few (IntHout et al., 2015). Hence, large, multi-center trials with improved study designs and implementation are required to validate our findings.

Finally,based on the GRADE method of assessing evidence, most of our results were considered to be of very low to moderate quality. Hence, additional high-quality clinical studies are required to support the rational treatment and application of CHM in pneumonia convalescence children. In addition, the evaluation of the total effective rate is often subjective. Also, a unified clinical expert consensus and diagnostic standards are required.

## 5 Conclusion

Our meta-analysis suggested that compared with WM alone, CHM combined with WM or CHM alone was more effective for the treatment of pneumonia convalescence in children. However, large, multi-center studies with well-thought-out study designs are required to demonstrate the efficacy of CHM on pneumonia convalescence in children.

## Data Availability

The original contributions presented in the study are included in the article/Supplementary Material; further inquiries can be directed to the corresponding author.
