# Supplementary material for: Efficacy and safety of Chinese herbal medicine for pneumonia convalescence in children: A systematic review and meta-analysis
Source: Front Pharmacol. 2022 Sep 2;13:956736. doi: 10.3389/fphar.2022.956736 (PMC9479002; doi:10.3389/fphar.2022.956736)
Supplement: Supplementary file 1 [file DataSheet1.docx]

Supplementary Material

# Supplementary Data

**Table of contents**

[1 Supplementary Data 1](#_Toc109653944)

[2 Supplementary File S1. Search Strategy 2](#_Toc109653945)

[3 Supplementary File S2. The detailed composition information of CHM 5](#_Toc109653946)

[4 Supplementary File S3. Results of subgroup analysis 10](#_Toc109653947)

[5 Supplementary File S4. Sensitivity analysis 14](#_Toc109653948)

[6 Supplementary File S5. PRISMA Checklist 15](#_Toc109653949)

# Supplementary File S1. Search Strategy

**CNKI**

(SU = '肺炎恢复期' OR SU='肺炎喘嗽恢复期' ) AND (SU='儿童' OR SU='小儿' OR SU='青少年' ) AND ( FT='中医' OR FT='中药' OR FT='中医药' OR FT='中西医' OR FT='中成药' OR FT='汤' OR FT='片' OR FT='丸' OR FT='散' OR FT='胶囊' OR FT='颗粒' OR FT='水' OR FT='液' OR FT='合剂' OR FT='注射液') AND (FT='随机')

n= 84

**VIP**

((M=肺炎恢复期 OR 肺炎喘嗽恢复期 ) OR (R=肺炎恢复期 OR 肺炎喘嗽恢复期 )) AND ((U=中医 OR 中药 OR 中西医OR中成药 OR 汤 OR 片 OR 丸 OR 散 OR 胶囊 OR 颗粒 OR水 OR液 OR 合剂 OR 注射液 ) OR (R=中医 OR 中药 OR 中西医OR中成药 OR 汤 OR 片 OR 丸 OR 散 OR 胶囊 OR 颗粒 OR水 OR液 OR 合剂 OR 注射液)) AND ((M=儿童 OR 小儿OR 青少年) OR (R=儿童 OR 小儿OR 青少年 )) AND ((U=随机 ) OR (R=随机 ))

n=12

**Wanfang**

全部:(( "肺炎恢复期" OR "肺炎喘嗽恢复期") AND ( "中医" OR "中药" OR "中西医" OR "中成药" OR "汤" OR "片" OR "丸" OR "散" OR "胶囊" OR "颗粒" OR "水" OR "液" OR "合剂" OR "注射液" ) AND ("儿童" OR"小儿" OR "青少年" )) AND ("随机" )

n=66

**CBM**

(("肺炎恢复期"[全部字段:智能] OR "肺炎喘嗽恢复期"[全部字段:智能] )) AND(( "中医"[全部字段:智能] OR "中药"[全部字段:智能] OR "中西医"[全部字段: 智能] OR ("中成药"[常用字段:智能] OR "汤"[全部字段:智能]) OR "片"[全部字段:智能] OR "丸"[全部字段:智能] OR "散"[全部字段:智能] OR "胶囊"[全部字段:智能] OR "颗粒"[全部字段:智能] OR "水"[全部字段:智能] OR"液"[全部字段:智能] OR "合剂"[全部字段:智能] OR "注射液"[全部字段:智能]))AND ("儿童"[全部字段:智能] OR "小儿"[全部字段:智能] OR "青少年"[全部字段:智能]) AND ("随机"[全部字段:智能])

n=56

**PubMed/Medline**

(((((pneumonia convalescence) OR (pneumonia in recovery period)) AND (((((children) OR (child)) OR (adolescent)) OR (teenager)) OR (youth))) AND (((((Chinese medicine) OR (TCM)) OR (Medicine, Chinese Traditional)) OR (Traditional Chinese Medicine)) OR (integrated Chinese and western medicine))) AND (((((herbal medicine) OR (herbalism)) OR (injection)) OR (decoction)) OR (chinese patent medicine))) AND (((((Random) OR (randomized controlled trial)) OR (RCT)) OR (randomized)) OR (Randomly))

n=4

**Embase**

#6. #1 AND #2 AND #3 AND #4 AND #5 n= 0

#5. random:ti,ab,kw OR 'randomized controlled 1,594,354 15 Dec 2021

trial':ti,ab,kw OR rct:ti,ab,kw OR

randomized:ti,ab,kw OR randomly:ti,ab,kw

#4. 'herbal medicine':ti,ab,kw OR herbalism:ti,ab,kw 714,963 15 Dec 2021

OR injection:ti,ab,kw OR decoction:ti,ab,kw OR

'chinese patent medicine':ti,ab,kw

#3. 'chinese medicine':ti,ab,kw OR tcm:ti,ab,kw OR 48,592 15 Dec 2021

'medicine, chinese traditional':ti,ab,kw OR

'traditional chinese medicine':ti,ab,kw OR

('integrated chinese':ti,ab,kw AND 'western

medicine':ti,ab,kw)

#2. pneumonia AND convalescence OR (pneumonia AND in 2,991 15 Dec 2021

AND recovery AND period)

#1. children:ti,ab,kw OR child:ti,ab,kw OR 1,985,318 15 Dec 2021

adolescent:ti,ab,kw OR teenager:ti,ab,kw OR

youth:ti,ab,kw

**Cochrane Library**

Date Run: 12/12/2021 15:33:21

#1 (pneumonia convalescence) OR (pneumonia in recovery period)ti,ab,kw

#2 ((children) OR (child) OR (adolescent) OR (teenager) OR (youth)):ti,ab,kw (Word variations have been searched)

#3 ((Chinese medicine) OR (TCM) OR (Medicine, Chinese Traditional) OR (Traditional Chinese Medicine) OR (integrated Chinese and western medicine)):ti,ab,kw (Word variations have been searched)

#4 ((herbal medicine) OR (herbalism) OR (injection) OR (decoction) OR (chinese patent medicine)):ti,ab,kw (Word variations have been searched)

#5 ((Random) OR (randomized controlled trial) OR (RCT) OR (randomized) OR (Randomly)):ti,ab,kw (Word variations have been searched)

#6 #1 and #2 and #3 and #4 and #5

n=0

# Supplementary File S2. The detailed composition information of CHM

| Study ID | Name of CHM | Composition of CHM |
| --- | --- | --- |
| Cui an Yi, 2009 | Liujunzi decoction | *Codonopsis pilosula (Franch.) Nannf., Atractylodes macrocephala Koidz., Smilax glabra Roxb., Citrus × aurantium L., Pinellia ternata (Thunb.) Makino, Glycyrrhiza glabra L.* |
| Du et al., 2017 | Yupingfeng granule | *Astragalus mongholicus Bunge, Saposhnikovia divaricata (Turcz. ex Ledeb.) Schischk., Atractylodes macrocephala Koidz.* |
| Gong and Guo, 2010 | Self-made formula | *Trichosanthes kirilowii Maxim., Pinellia ternata (Thunb.), Rumex crispus L., Smilax glabra Roxb., Kitagawia praeruptora (Dunn) Pimenov, Citrus × aurantium L., Scutellaria baicalensis Georgi, Codonopsis pilosula (Franch.) Nannf.* |
| Guo et al., 2015 | Self-made formula | *Pseudostellariae Radix, Prunus armeniaca L., Kitagawia praeruptora (Dunn) Pimenov, Fritillaria cirrhosa D.Don, Platycodon grandifloras (Jacq.) A.DC., Citrus × aurantium L., Descurainia sophia (L.) Webb ex Prantl, Massa Medicata Fermentata, Vincetoxicum stauntonii (Decne.) C.Y.Wu & D.Z.Li, Perilla frutescens (L.) Britton, Perilla frutescens (L.) Britton, Coix lacryma-jobi var. ma-yuen (Rom.Caill.) Stapf, Atractylodes macrocephala Koidz., Hordeum vulgare L.* |
| Guo et al., 2020 | Qingfeitongluo decoction | *Glycyrrhiza glabra L., Prunus armeniaca L., Prunus persica (L.) Batsch, Lycium barbarum L., Morus alba L., Ardisia japonica (Thunb.) Blume, Ephedra Herba, Pheretima, Perilla frutescens (L.) Britton* |
| Han, 2009 | Sha sheng mai dong Decoction | *Adenophora triphylla (Thunb.) A.DC., Ophiopogon japonicus (Thunb.) Ker Gawl., Smilax glabra Roxb., Polygonatum odoratum (Mill.) Druce, Fritillaria cirrhosa D.Don, Lycium barbarum L., Perilla frutescens (L.) Britton* |
| Hou, 2016 | Liujunzi decoction | *Codonopsis pilosula (Franch.) Nannf., Atractylodes macrocephala Koidz., Smilax glabra Roxb., Citrus × aurantium L., Pinellia ternata (Thunb.) Makino, Glycyrrhiza glabra L.* |
| Huang et al., 2013 | Self-made formula | *Bupleurum chinense DC., Astragalus mongholicus Bunge, Atractylodes macrocephala Koidz., Codonopsis pilosula (Franch.) Nannf., Smilax glabra Roxb., Citrus × aurantium L., Saposhnikovia divaricata (Turcz. ex Ledeb.) Schischk., Paeonia lactiflora Pall., Citrus Reticulata, Glycyrrhiza glabra L.* |
| Liang and He, 2018 | Huoxuehuayu decoction | *(1)Rehmannia glutinosa (Gaertn.) DC., Scrophularia ningpoensis Hemsl., Astragalus mongholicus Bunge, Ophiopogon japonicus (Thunb.) Ker Gawl., Schisandra chinensis (Turcz.) Baill., Prunus persica (L.) Batsch, Glycyrrhiza glabra L., Scutellaria baicalensis Georgi, Fritillaria cirrhosa D.Don, Tussilago farfara L. (2)Codonopsis pilosula (Franch.) Nannf., Atractylodes macrocephala Koidz., Platycodon grandifloras (Jacq.) A.DC., Salvia miltiorrhiza Bunge, Glycyrrhiza glabra L., Fritillaria cirrhosa D.Don* |
| Li et al., 2019 | XiaoErFeiKe granule | *Panax ginseng C.A.Mey., Smilax glabra Roxb., Atractylodes macrocephala Koidz., Citrus × aurantium L., Galli gigeriae endothelium corneum, Rheum palmatum L., Carapax Trionycis, Lycium barbarum L., Adenophora triphylla (Thunb.) A.DC., Glycyrrhiza glabra L., Artemisia annua L., Ophiopogon japonicus (Thunb.) Ker Gawl., Neolitsea cassia (L.) Kosterm.,* *Zingiber officinale Roscoe, Aconitum carmichaeli Debeaux, Trichosanthes kirilowii Maxim., Tussilago farfara L., Aster tataricus L.f., Morus alba L., Arisaema erubescens (Wall.) Schott, Astragalus mongholicus Bunge, Lycium chinense Mill.* |
| Liu et al., 2009 | Addition of Six Junzi Decoction | *Codonopsis pilosula (Franch.) Nannf., Atractylodes macrocephala Koidz., Smilax glabra Roxb., Astragalus mongholicus Bunge, Citrus × aurantium L., Citrus Reticulata, Arum Ternatum Thunb., Platycodon grandifloras (Jacq.) A.DC., Aster tataricus L.f., Angelica sinensis (Oliv.) Diels, Salvia miltiorrhiza Bunge, Glycyrrhiza glabra L.* |
| Liu et al., 2014 | Yiqijianpihuoxue decoction | *Astragalus mongholicus Bunge, Atractylodes macrocephala Koidz., Saposhnikovia divaricata (Turcz. ex Ledeb.) Schischk., Arum ternatum Thunb., Citrus × aurantium L., Smilax glabra Roxb., Salvia miltiorrhiza Bunge, Prunus persica (L.) Batsch, Glycyrrhiza glabra L.* |
| Lu et al., 2013 | Addition of yupingfeng oral liquid and Xingbei expectorant cough oral liquid | NR |
| Tian, 2009 | Yiqihuoxue decoction | *Astragalus mongholicus Bunge, Atractylodes macrocephala Koidz., Arum ternatum Thunb., Citrus × aurantium L., Smilax glabra Roxb., Saposhnikovia divaricata (Turcz. ex Ledeb.) Schischk., Reynoutria japonica Houtt., Salvia miltiorrhiza Bunge, Prunus persica (L.) Batsch, Glycyrrhiza glabra L.* |
| Wang and Hu, 2019 | XiaoErFeiKe granule | *Panax ginseng C.A.Mey., Smilax glabra Roxb., Atractylodes macrocephala Koidz., Citrus × aurantium L., Galli gigeriae endothelium corneum, Rheum palmatum L., Carapax Trionycis, Lycium barbarum L., Adenophora triphylla (Thunb.) A.DC., Glycyrrhiza glabra L., Artemisia annua L., Ophiopogon japonicus (Thunb.) Ker Gawl., Neolitsea cassia (L.) Kosterm., Zingiber officinale Roscoe, Aconitum carmichaeli Debeaux, Trichosanthes kirilowii Maxim., Tussilago farfara L., Aster tataricus L.f., Morus alba L., Arisaema erubescens (Wall.) Schott, Astragalus mongholicus Bunge, Lycium chinense Mill.* |
| Ye and Zhen, 2016 | XiaoErFeiKe granule | *Panax ginseng C.A.Mey., Smilax glabra Roxb., Atractylodes macrocephala Koidz., Citrus × aurantium L., Galli gigeriae endothelium corneum, Rheum palmatum L., Carapax Trionycis, Lycium barbarum L., Adenophora triphylla (Thunb.) A.DC., Glycyrrhiza glabra L., Artemisia annua L., Ophiopogon japonicus (Thunb.) Ker Gawl., Neolitsea cassia (L.) Kosterm., Zingiber officinale Roscoe, Aconitum carmichaeli Debeaux, Trichosanthes kirilowii Maxim., Tussilago farfara L., Aster tataricus L.f., Morus alba L., Arisaema erubescens (Wall.) Schott, Astragalus mongholicus Bunge, Lycium chinense Mill.* |
| Yu and Gao, 2010 | Danshen injection | *Salvia miltiorrhiza Bunge* |
| Zhang, 2018 | Lin gui zhi ke Combination | *Smilax glabra Roxb., Neolitsea cassia (L.) Kosterm., Astragalus mongholicus Bunge, Zingiber officinale Roscoe, Atractylodes macrocephala Koidz., Atractylodes lancea (Thunb.) DC., Salvia miltiorrhiza Bunge, Prunus persica (L.) Batsch, Arum Ternatum Thunb., Citrus × aurantium L., Stemona tuberosa Lour., Bambusa tuldoides Munro, Trichosanthes kirilowii Maxim., Glycyrrhiza glabra L.* |
| Zhang, 2020 | Erchen decoction pr xiaoqinglong decoction | *Arum ternatum Thunb., Citrus × aurantium L., Smilax glabra Roxb., Glycyrrhiza glabra L., Neolitsea cassia (L.) Kosterm., Paeonia lactiflora Pall., Ephedra sinica Stapf, Zingiber officinale Roscoe, Asarum heterotropoides F.Schmidt, Glycyrrhiza glabra L., Arum ternatum Thunb., Schisandra chinensis (Turcz.) Baill., Ziziphus jujuba Mill.* |
| Zhu et al., 2020 | Ren shen wu wei zi Decoction or Sha sheng mai dong Decoction | *(1)Codonopsis pilosula (Franch.) Nannf., Smilax glabra Roxb., Atractylodes macrocephala Koidz., Glycyrrhiza glabra L., Schisandra chinensis (Turcz.) Baill., Stemona tuberosa Lour., Citrus × aurantium L., Arum ternatum Thunb. (2)Adenophora triphylla (Thunb.) A.DC., Ophiopogon japonicus (Thunb.) Ker Gawl., Polygonatum odoratum (Mill.) Druce, Trichosanthes kirilowii Maxim., Morus alba L., Tussilago farfara L., Glycyrrhiza glabra L., Lablab purpureus subsp. purpureus, Stemona tuberosa Lour.* |

Note: NR, NR, not reported; The species names were validated taxonomically using the Kew Medicinal Plant Names Services (MPNS) (<https://mpns.science.kew.org>). However, the three drugs are non-plant drugs, and their pharmacological studies have been reported. We supplemented their species names by referring to the literature.

1.Massa Medicata Fermentata (Liu Shenqu) is a natural fermentation product of Prunus armeniaca L., Vigna umbellata Thunb., Artemisia annua L., Polygonum hydropiper L., Xanthium strumarium L. and wheat bran or flour (Triticum aestivum L.)(Liu et al., 2021).

2.Galli gigeriae endothelium corneum (GGEC), the dried inner wall of the Gallus gallus domesticus Brisson as a chicken by-product(Li et al., 2021).

3.Carapax Trionycis, as a traditional Chinese medicine, originated from the shell of Trionyx sinensis Wiegmann(Tang et al., 2013).

# Supplementary File S3. Results of subgroup analysis

2.1 Subgroup analysis of the total clinical effective rate according to the therapy method (CHM+WM vs. WM, and CHM vs. WM).


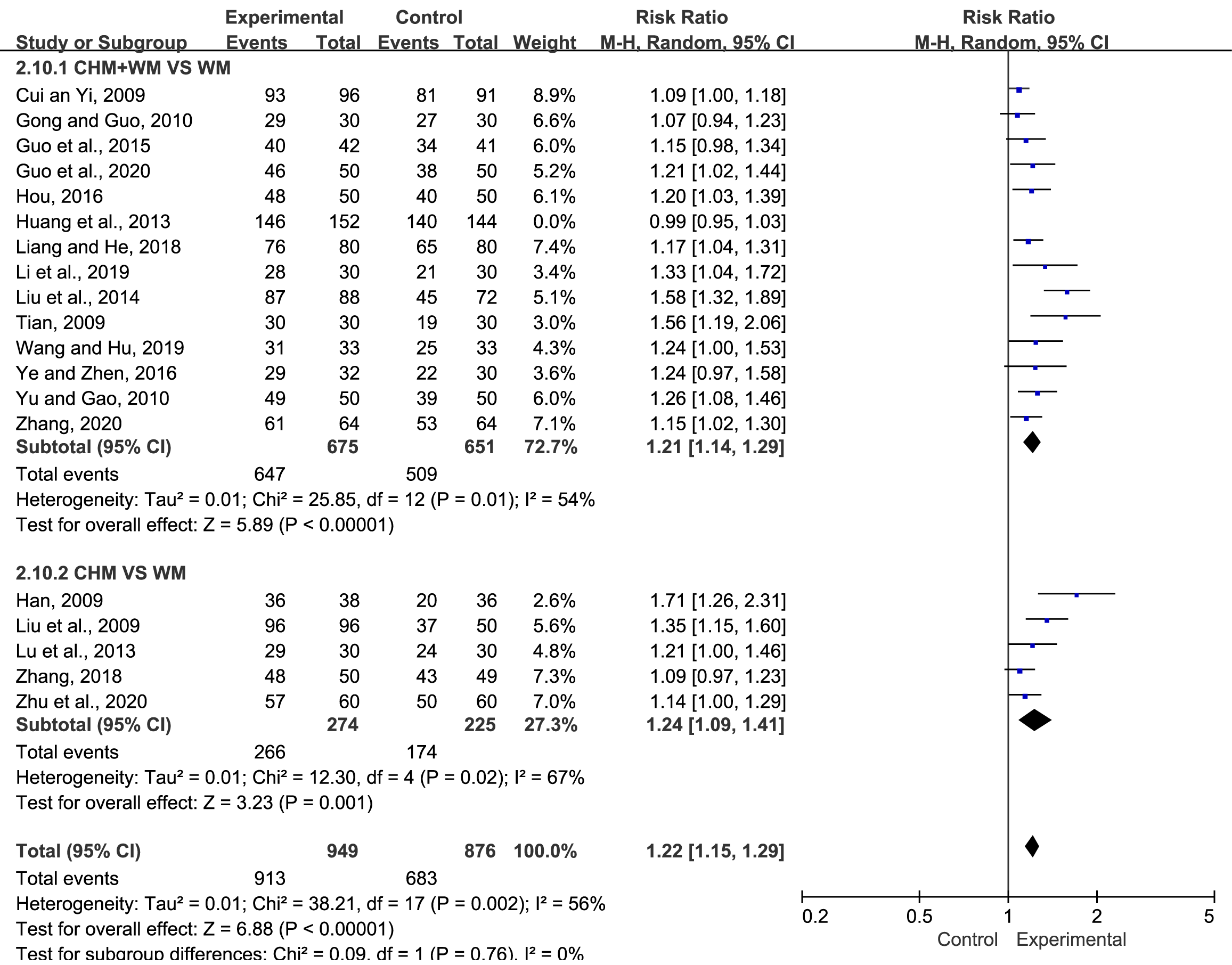


2.2 Subgroup analysis of the total clinical effective rate according to the treatment course.
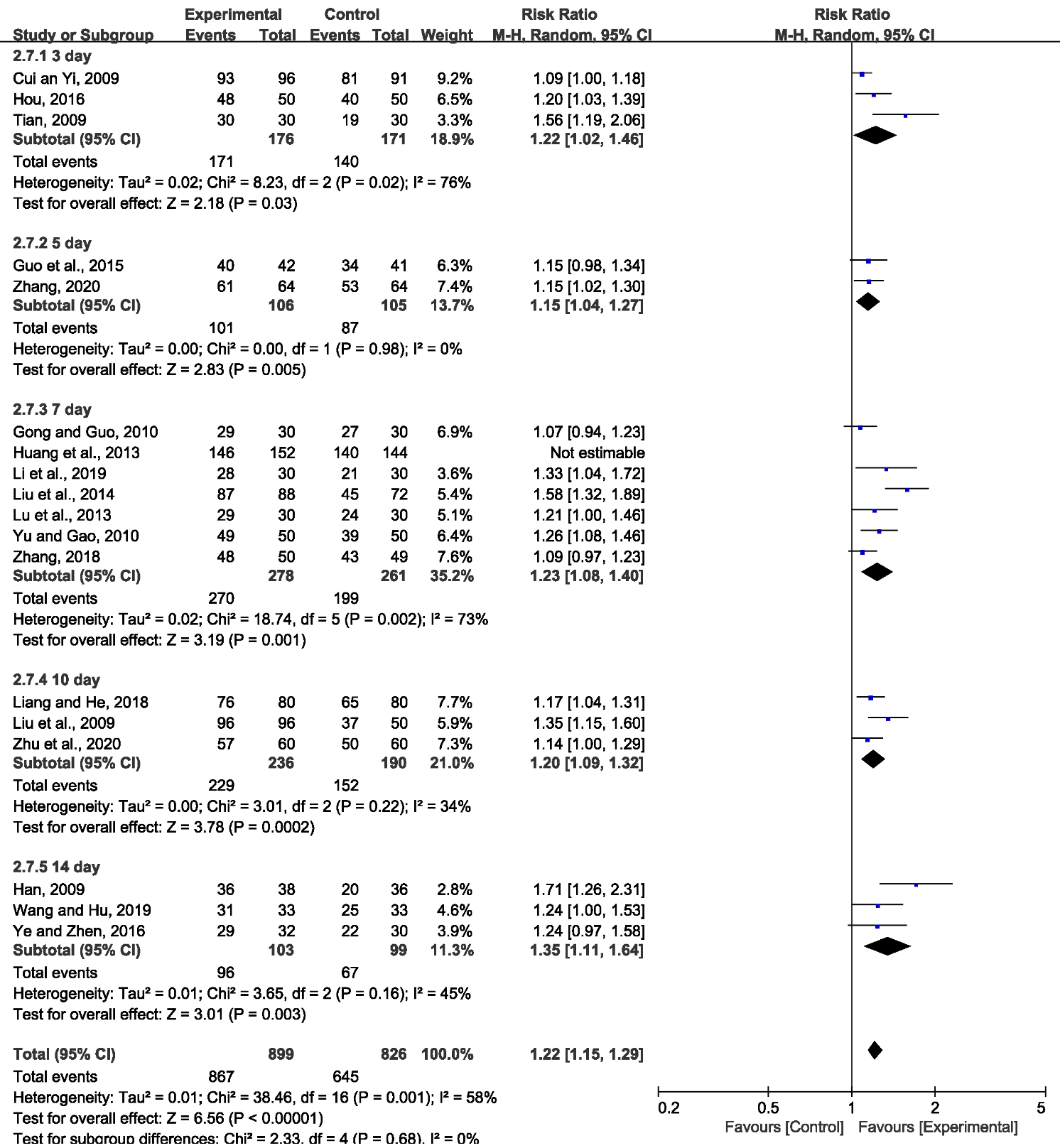


2.3 Subgroup analysis of the total clinical effective rate according to the efficacy judgment standard.
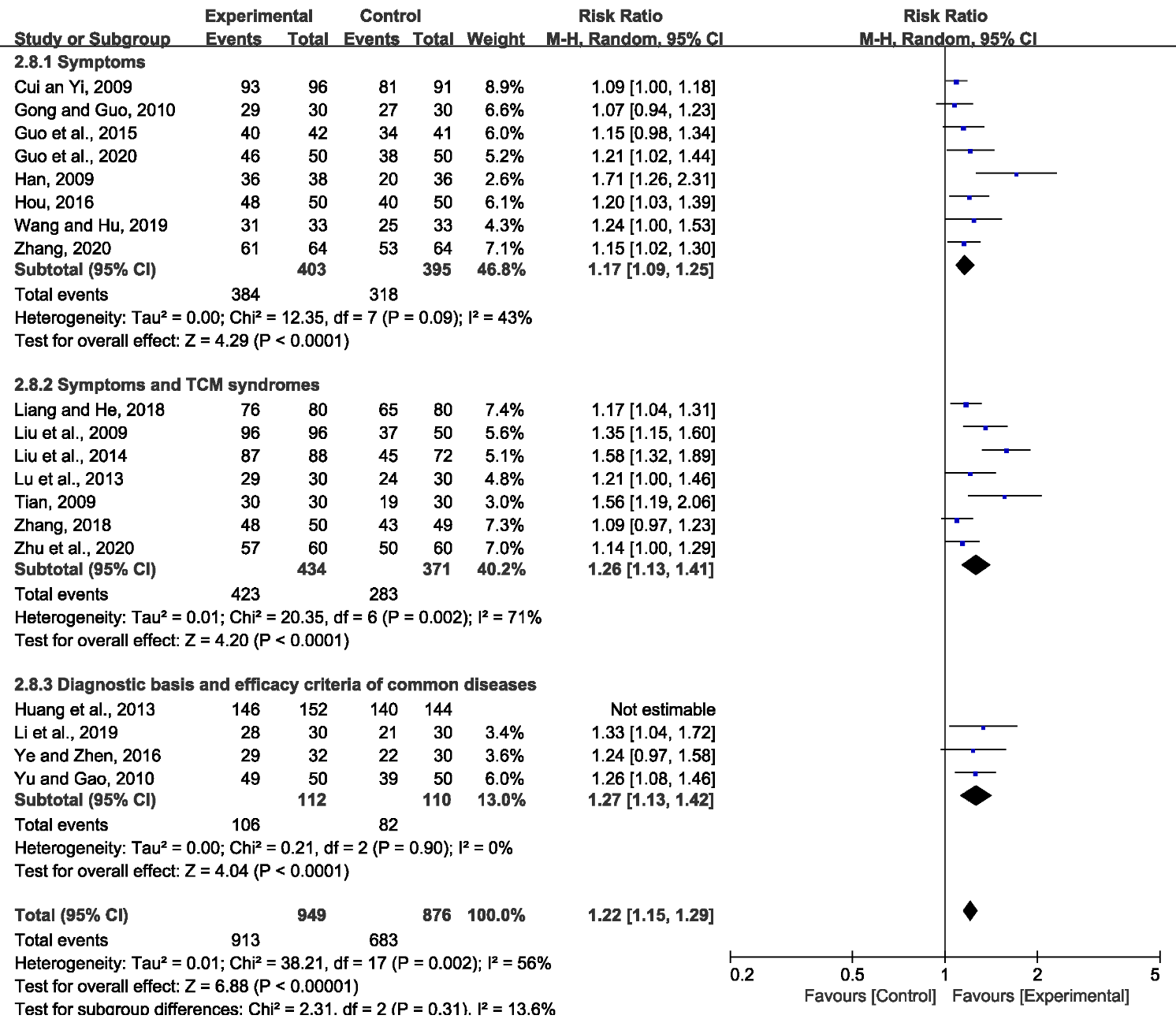


2.4 Subgroup analysis of the total clinical effective rate according to the diagnostic criteria standard.
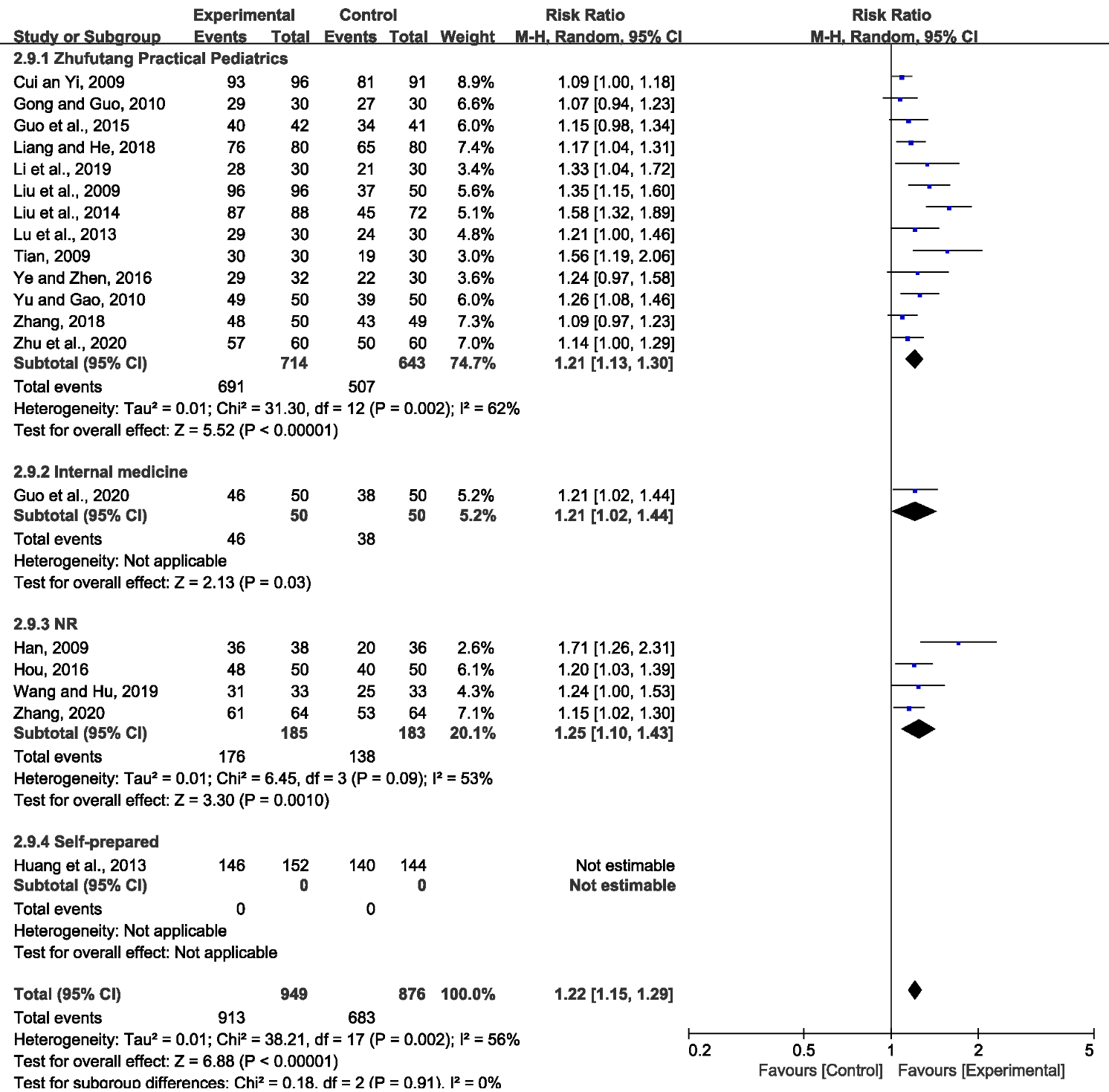


# Supplementary File S4. Sensitivity analysis


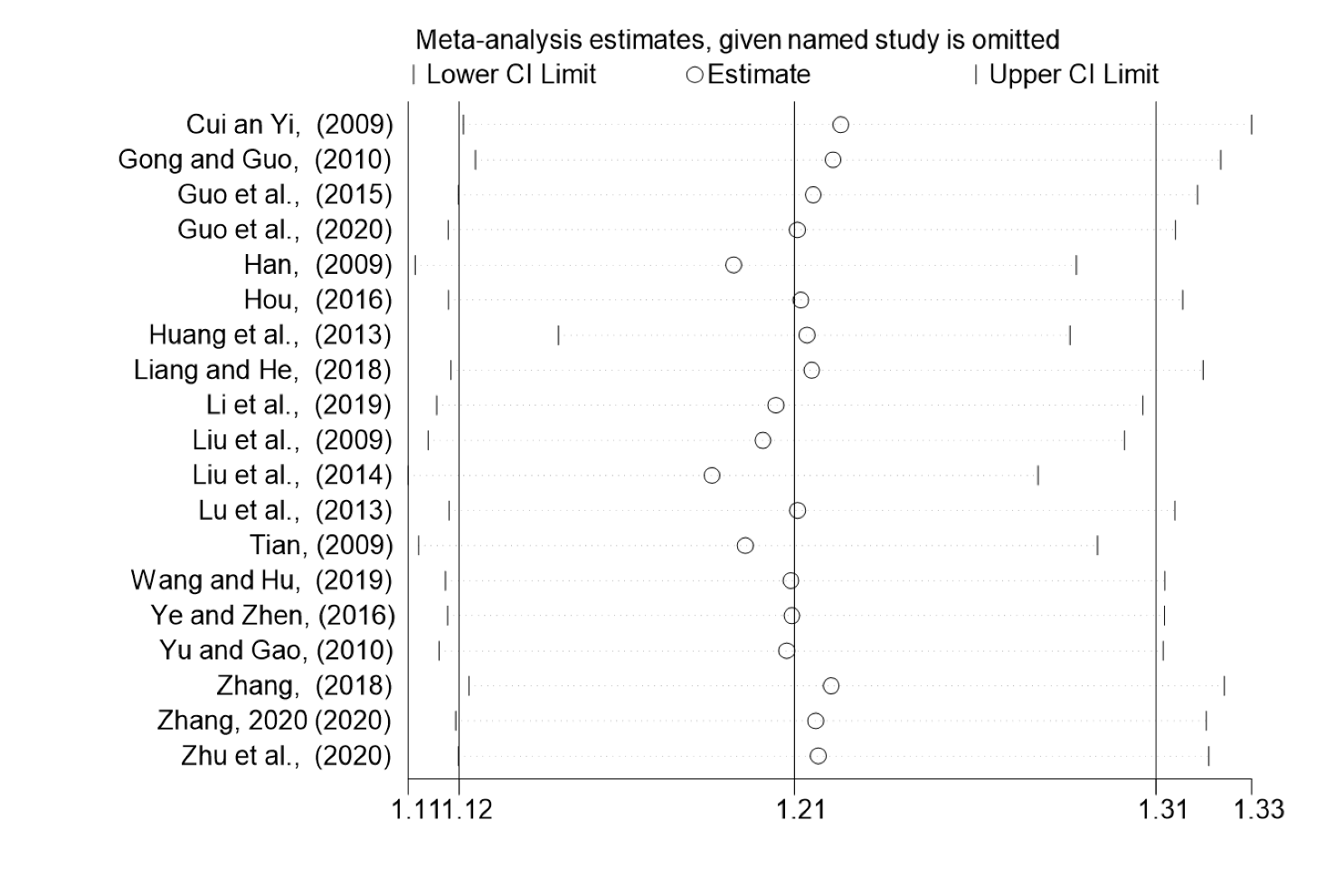


Li, S., Zheng, M., Zhang, Z., Peng, H., Dai, W., and Liu, J. (2021). Galli gigeriae endothelium corneum: its intestinal barrier protective activity in vitro and chemical composition. *Chin Med* 16(1)**,** 22. doi: 10.1186/s13020-021-00432-3.

Liu, Y., Liao, W., Liu, X., Hu, Y., Zhu, X., Ju, L., et al. (2021). Digestive promoting effect and mechanism of Jiao Sanxian in rats. *J Ethnopharmacol* 278**,** 114334. doi: 10.1016/j.jep.2021.114334.

Tang, Y., Hu, C., and Liu, Y. (2013). Effect of bioactive peptide of Carapax Trionycis on TGF-beta1-induced intracellular events in hepatic stellate cells. *J Ethnopharmacol* 148(1)**,** 69-73. doi: 10.1016/j.jep.2013.03.067.

| **Section and Topic** | **Item #** | **Checklist item** | **Location where item is reported** |
| --- | --- | --- | --- |
| **TITLE** | | |  |
| Title | 1 | Identify the report as a systematic review. | P1 |
| **ABSTRACT** | | |  |
| Abstract | 2 | See the PRISMA 2020 for Abstracts checklist. | P1-P2 |
| **INTRODUCTION** | | |  |
| Rationale | 3 | Describe the rationale for the review in the context of existing knowledge. | P2-P3 |
| Objectives | 4 | Provide an explicit statement of the objective(s) or question(s) the review addresses. | P2-P3 |
| **METHODS** | | |  |
| Eligibility criteria | 5 | Specify the inclusion and exclusion criteria for the review and how studies were grouped for the syntheses. | P3-P4 |
| Information sources | 6 | Specify all databases, registers, websites, organisations, reference lists and other sources searched or consulted to identify studies. Specify the date when each source was last searched or consulted. | P3 |
| Search strategy | 7 | Present the full search strategies for all databases, registers and websites, including any filters and limits used. | P3 Supplementary file S1 |
| Selection process | 8 | Specify the methods used to decide whether a study met the inclusion criteria of the review, including how many reviewers screened each record and each report retrieved, whether they worked independently, and if applicable, details of automation tools used in the process. | P4-P5 |
| Data collection process | 9 | Specify the methods used to collect data from reports, including how many reviewers collected data from each report, whether they worked independently, any processes for obtaining or confirming data from study investigators, and if applicable, details of automation tools used in the process. | P4-P5 |
| Data items | 10a | List and define all outcomes for which data were sought. Specify whether all results that were compatible with each outcome domain in each study were sought (e.g. for all measures, time points, analyses), and if not, the methods used to decide which results to collect. | P4-P5 |
|  | 10b | List and define all other variables for which data were sought (e.g. participant and intervention characteristics, funding sources). Describe any assumptions made about any missing or unclear information. | P4,P14-P15 |
| Study risk of bias assessment | 11 | Specify the methods used to assess risk of bias in the included studies, including details of the tool(s) used, how many reviewers assessed each study and whether they worked independently, and if applicable, details of automation tools used in the process. | P4-P5 |
| Effect measures | 12 | Specify for each outcome the effect measure(s) (e.g. risk ratio, mean difference) used in the synthesis or presentation of results. | P5 |
| Synthesis methods | 13a | Describe the processes used to decide which studies were eligible for each synthesis (e.g. tabulating the study intervention characteristics and comparing against the planned groups for each synthesis (item #5)). | P4-P5 and P14-P15 |
|  | 13b | Describe any methods required to prepare the data for presentation or synthesis, such as handling of missing summary statistics, or data conversions. | P4-P5 |
|  | 13c | Describe any methods used to tabulate or visually display results of individual studies and syntheses. | P4-P5 |
|  | 13d | Describe any methods used to synthesize results and provide a rationale for the choice(s). If meta-analysis was performed, describe the model(s), method(s) to identify the presence and extent of statistical heterogeneity, and software package(s) used. | P5 |
|  | 13e | Describe any methods used to explore possible causes of heterogeneity among study results (e.g. subgroup analysis, meta-regression). | P5 |
|  | 13f | Describe any sensitivity analyses conducted to assess robustness of the synthesized results. | P5 |
| Reporting bias assessment | 14 | Describe any methods used to assess risk of bias due to missing results in a synthesis (arising from reporting biases). | P5 |
| Certainty assessment | 15 | Describe any methods used to assess certainty (or confidence) in the body of evidence for an outcome. | P5 |
| **RESULTS** | | |  |
| Study selection | 16a | Describe the results of the search and selection process, from the number of records identified in the search to the number of studies included in the review, ideally using a flow diagram. | P5 and Figure 1 |
|  | 16b | Cite studies that might appear to meet the inclusion criteria, but which were excluded, and explain why they were excluded. | P5 and Figure 1 |
| Study characteristics | 17 | Cite each included study and present its characteristics. | P6 |
| Risk of bias in studies | 18 | Present assessments of risk of bias for each included study. | P6 |
| Results of individual studies | 19 | For all outcomes, present, for each study: (a) summary statistics for each group (where appropriate) and (b) an effect estimate and its precision (e.g. confidence/credible interval), ideally using structured tables or plots. | P6-P8 |
| Results of syntheses | 20a | For each synthesis, briefly summarise the characteristics and risk of bias among contributing studies. | P6-P8 |
|  | 20b | Present results of all statistical syntheses conducted. If meta-analysis was done, present for each the summary estimate and its precision (e.g. confidence/credible interval) and measures of statistical heterogeneity. If comparing groups, describe the direction of the effect. | P6-P8 |
|  | 20c | Present results of all investigations of possible causes of heterogeneity among study results. | P6-P8 and P11 |
|  | 20d | Present results of all sensitivity analyses conducted to assess the robustness of the synthesized results. | P6-P8 |
| Reporting biases | 21 | Present assessments of risk of bias due to missing results (arising from reporting biases) for each synthesis assessed. | P8 |
| Certainty of evidence | 22 | Present assessments of certainty (or confidence) in the body of evidence for each outcome assessed. | P8 |
| **DISCUSSION** | | |  |
| Discussion | 23a | Provide a general interpretation of the results in the context of other evidence. | P9-P10 |
|  | 23b | Discuss any limitations of the evidence included in the review. | P11-P12 |
|  | 23c | Discuss any limitations of the review processes used. | P11-P12 |
|  | 23d | Discuss implications of the results for practice, policy, and future research. | P11 |
| **OTHER INFORMATION** | | |  |
| Registration and protocol | 24a | Provide registration information for the review, including register name and registration number, or state that the review was not registered. | P3 |
|  | 24b | Indicate where the review protocol can be accessed, or state that a protocol was not prepared. | P3 |
|  | 24c | Describe and explain any amendments to information provided at registration or in the protocol. | P3 |
| Support | 25 | Describe sources of financial or non-financial support for the review, and the role of the funders or sponsors in the review. | P17 |
| Competing interests | 26 | Declare any competing interests of review authors. | P17 |
| Availability of data, code and other materials | 27 | Report which of the following are publicly available and where they can be found: template data collection forms; data extracted from included studies; data used for all analyses; analytic code; any other materials used in the review. | P21 |

# Supplementary File S5. PRISMA Checklist

*From:*  Page MJ, McKenzie JE, Bossuyt PM, Boutron I, Hoffmann TC, Mulrow CD, et al. The PRISMA 2020 statement: an updated guideline for reporting systematic reviews. BMJ 2021;372:n71. doi: 10.1136/bmj.n71

For more information, visit: <http://www.prisma-statement.org/>
